# The Use of Robots in the Workplace: Conclusions from a Health Promoting Intervention Using Social Robots

**DOI:** 10.1007/s12369-023-01000-5

**Published:** 2023-04-24

**Authors:** Sara L. Lopes, Aristides I. Ferreira, Rui Prada

**Affiliations:** 1 Instituto Universitário de Lisboa (Iscte-IUL) & Business Research Unit (BRU-IUL), Iscte-IUL, Lisbon, Portugal; 2 INESC-ID & Instituto Superior Técnico, Universidade de Lisboa, Lisbon, Portugal

**Keywords:** Human–robot interaction, Workplace intervention, Health intervention

## Abstract

Workplace wellness programs constitute a preventive measure to help avoid healthcare costs for companies, with additional benefits for employee productivity and other organizational outcomes. Interventions using social robots may have some advantages over other conventional telemedicine applications, since they can deliver personalized feedback and counseling. This investigation focused on a health-promoting intervention within work environments, and compared the efficacy of the intervention on two distinct groups, one guided by a human agent and the other by a robot agent. Participants (n = 56) were recruited from two Portuguese organizations and led through eight sessions by the social agent, the goal being to encourage health behavior change and adoption of a healthier lifestyle. The results indicate that the group led by the robot agent revealed better post-intervention scores than the group led by the human agent, specifically with regard to productivity despite presenteeism and regard of their level of mental well-being. No effects were found concerning the work engagement level of participants in either group. By demonstrating the potential of using social robots to establish therapeutic and worth relationships with employees in their workplaces, this study provides interesting new findings that contribute to the literature on health behavior change and human–robot interaction.

## Introduction

The last few years have been marked by the emergence and establishment of Industry 4.0 and with it, several technological advances that are making robotics more and more ubiquitous in everyday life. This has opened up new possibilities and applications regarding the use of social robots, including in health applications [1]. Social robots can be useful in a variety of ways and settings, such as in hospitals, in healthcare and therapy, as well as for promoting the adoption of health behaviors [2]. What is more, social robots could also have some advantages over other telemedicine applications, since they can deliver personalized behavioral change interventions [3], and may be able to meet individuals’ needs and help them achieve their personal goals. Workplace wellness and health promotion programs constitute preventive measures that can help companies to avoid healthcare costs, increase productivity, and enhance other organizational outcomes, as well as improving employees’ well-being [4]. Such programs, based on health behavior change, are developed to help individuals adjust their health behaviors and, ultimately, to adopt healthier lifestyles [5]. In general, workplace interventions focus on chronic health conditions and their associated multiple health risk factors [6]. Some of these conditions, such as cardiovascular disease, diabetes, high cholesterol, smoking, stress and sedentarism, are among the most common and preventable health concerns, since they are directly related to lifestyle risk factors [6]. However, most workplace intervention programs are not theory-driven and very few provide personalized feedback [7]. Many are based on the Health Action Process Approach (HAPA), which is a theoretical framework designed to understand health behavior change [8]. Taking a broader view, health behavior change programs have been linked to improvements in workers’ satisfaction, lower absenteeism, promoting a sense of community, and improvements in long-term health [9].

Workplace health promotion programs are also linked to lower levels of presenteeism [10]. Presenteeism is the term used to describe the act, or culture, of employees working while not in full health [11], which often has hidden costs for organizations. Several approaches have been linked to presenteeism, such as the job demands-resources (JD-R) model [12]. This model states that presenteeism may arise because of a lack of resources in the workplace, causing stress to employees and forcing them to deal with extreme job demands [13]. As an innovative solution to address the issue of increased presenteeism, it has been suggested that intervention programs should focus on helping workers learn how to cope better with job demands while, at the same time, ensuring adequate workplace resources [13]. With this in mind, it is possible that robots could be used as an important workplace resource to reduce the demands that contribute to presenteeism and help employees to adopt healthier behaviors.

Past research has already endeavored to determine the possibilities of social robotics helping to prevent presenteeism behaviors. The work of [14] acknowledged that social robots have the potential to be more readily accepted than a human colleague in situations where individuals are working ill. Consequently, they can assist workers whose health status makes it difficult for them to accomplish work demands.

Although some health conditions can be improved by health behavior change, that behavioral change can be difficult to maintain, especially over the long term, as reported in previous research [5, 7, 9, 36]. When compared with other telemedicine applications, interventions using social robots may prove to provide greater benefits since they promote human–robot interaction, where social robots can ask questions, give feedback, and can offer advice that is personalized to the user [1, 19, 22, 25]. However, these advantages may only occur if interactions with the social robot agent are incorporated in several sessions over a longer period of time.

Bearing this in mind, and with presenteeism and the theoretical background of the HAPA in the frame, the purpose of this present investigation was to develop a longitudinal health promoting intervention using both a social robot and a human in the roles of health-promoting agents. We focused on four groups of lifestyle risk factors: physical inactivity, nutrition habits, tobacco consumption and stress management. These are the ones related to a higher incidence of chronic ill-health [6], besides also being those that are more prevalent in workplace scenarios [15]. We compared participants’ outcomes against a set of variables that were measured before the investigation (Time 1) and after the intervention with the social agent (Time 2) to assess the role of a robotic agent versus a human agent in a health-promoting intervention.

This research aims to contribute to presenteeism literature by using an intervention to show the impact that workplace illness has on productivity and other organizational outcomes, such as work engagement and role ambiguity. Moreover, we are also interested in contributing to the HAPA theoretical framework by providing an application of this model through a robotic agent, following previous work using HAPA-driven technological health interventions [16, 17]. This can also contribute to the JD-R model [12], by providing managers with an innovative solution for reducing the job demands associated with presenteeism. Finally, this work can contribute to our knowledge of the socio-cognitive aspects involved in interaction between humans and robots and to the field of human–robot interaction overall.

## Theoretical Background and Hypotheses

### Social Robots as Health-Promoting Agents

The use of social robots to provide guidance for health behavior change can offer greater flexibility than ‘normal’ telemedicine methods can [2]. This study follows the definition of a social robot given by the authors Bartneck and Forlizzi (p. 2) [18]: “a social robot is an autonomous or semi-autonomous robot that interacts and communicates with humans by following the behavioral norms expected by the people with whom the robot is intended to interact”. Social robots have been used to provide therapeutic assistance in healthcare for a wide range of users, from children to adults and even for the elderly [19, 20]. They can be used to motivate, coach, educate, provide feedback and social support, and thus improve compliance with health behavior change programs. Previous research has previously established that social robots can “humanely care” for people suffering from physical or mental problems [21]. In this way, since social robots can establish personalized and affective relationships with users, they can help them meet specific targets, such as mental and behavioral goals [2], and they can also help improve individuals’ quality of life [22]. Given the difficulties that organizations and public health systems encounter with regard to providing face-to-face individualized health counseling [23], using social robots may afford opportunities to improve access to health promotion programs and encourage health behavioral change [24].

The literature on human–robot interaction is rich in interventions showing that robots can be used to improve individuals’ psychological and physical health. A meta-analysis by Costescu et al. [22] revealed the medium-size significant effect of robot-enhanced therapy on improving behavioral and cognitive aspects for individuals involved in psychotherapy treatments. Another meta-analysis regarding the use of social robots in mental health and wellness interventions revealed significant differences in patients' mood and quality of life, after intervention with the robot [3].

Another investigation that used a social robot to provide personalized feedback for health promotion found that patients reported being just as comfortable discussing health-related topics with a robot as with a human [25]. Furthermore, a pilot randomized controlled trial found that people reduced the average number of high-calorie foods and drinks after completing an intervention with the robot, without any human involvement [26].

Despite these interesting findings, there are nevertheless still some gaps in the literature on human–robot interaction. First, most of the studies are published in robotics journals, where the focus is almost exclusively on the technical details of robots, rather than on the intervening variables and methodological details [22]. With the present investigation, we aim to emphasize some psychological and organizational aspects that can be relevant in the interaction between humans and robots. Moreover, we intend to make a comparison between two groups of participants, one where the intervention is led by a robotic agent and the other by a human agent. To our knowledge, although this constitutes an unusual methodological approach, it provides an important opportunity to shed light on the key differences between interaction with humans and interaction with robots. In line with that, the use of robots for therapy and counseling purposes has so far been limited to education and engagement concerning healthcare issues [24]. Dialogue and interaction between a human and a robot have not been a crucial focus, although recent investigations have demonstrated that individuals report feeling more comfortable discussing health issues with robots than with a human counselor, since robots are perceived as nonjudgmental [24]. In order, therefore, to thoroughly understand the effect this may have on health behavior change, this study centers on the dialogue and interaction between the participants and the robot agent. What is more, social robots might possess characteristics that could lead individuals to perform better in health-promoting interventions led by them rather than by human counselors.

The field of human–robot interaction has been guided by debates concerning the capability of humans to establish empathetic and trustworthy relationships with social robots [27]. These processes have been analyzed extensively in human–robot interaction research through socio-cognitive explanations, and a large body of research has found that people do empathize with and even trust robots [28, 29]. Moreover, social robots can also engage in actions to reduce individuals’ stress, in the form of social supportive behaviors [29]. This means that although robots do not possess a real consciousness, they can demonstrate empathetic and trustworthy behaviors [30]. This happens because humans apply the same socio-cognitive processes when interacting with social robots as when interacting with other humans [31, 32]. Therefore, interaction with social robots can be easy if social robots display rich social behavior and social feature levels similar to those of humans [33]. All of this provides further evidence to suggest that people feel more comfortable discussing health-related issues with robots than with human agents, since robots are considered to be more tolerant, nonjudgmental, and capable of demonstrating empathic behaviors [1].

However, the ethical issues raised by interactions between humans and robots have been repeatedly and rightly raised. It is up to businesses to take the first steps in ensuring that robots in the workplace truly help users and are not used solely to reduce an organization's operational costs. Employees need to feel that they can trust these robotic agents, in order for these types of interventions to produce positive outcomes [34]. Robotic systems are designed by humans, and the majority of social robots require human assistance, so this can inspire confidence that robots are not here to replace us, but to help and assist us [34]. Companies must also guarantee data use and privacy. Aside from that, organizations must recognize and accept that some employees may be afraid of interacting with social robots and would prefer only human interaction/collaboration. Nevertheless, prior research has already established that individuals are willing to accept social robots in their work environments [14, 25, 26].

Furthermore, little is known about the potential social robots have to improve the health and well-being of individuals in working populations (Sebo et al. [35]). As work environments are places where individuals spend the majority of their time, they are ideal settings for the application of human–robot interventions. Given the previously mentioned gaps in the literature and following recent recommendations concerning the use of robots in workplaces (e.g., [35]), this investigation focuses on the potential of using a social robot as a health-promoting agent within work environments. Ultimately, although most health behavior change interventions track health outcomes throughout multiple sessions or over time, this has not been the case with robotic interventions. These are usually developed in single-one contexts [25], which may lead to a dim view being taken of using robots as health-promoting agents. In this present intervention, we aimed to make it longitudinal, with multiple sessions and multiple interactions with the health promoting agent.

### The HAPA Model and Healthy Behaviors in the Workplace

The HAPA model, developed by Schwarzer [8, 36] suggests that the adoption, initiation, and maintenance of health behaviors result from a set of social-cognitive predictors that operate by translating intentions into behaviors [37]. The distinguishing aspect of this research resides in applying the HAPA model in an intervention delivered by an artificial intelligence machine, with the purpose of promoting healthier behaviors among employees. Although several other investigations have applied HAPA to health behavior change interventions, to the best of our knowledge none of those HAPA interventions have compared the efficacy of the intervention with a human agent versus a robot agent, much less with a set of individual and organizational variables. Our goal is to compare the role of a human agent and a robotic agent in a health-promoting intervention, by comparing participants’ results against a set of variables that were measured prior to the investigation (Time 1) and after the intervention (Time 2).

People often engage in lifestyle risk behaviors that can compromise their physical and psychological health [6]. Beyond the direct consequences for the individual, these behaviors impose a substantial burden on society’s health care resources [24] and, in turn, on organizations and companies. Workplace health programs, which are interventions designed to reduce health care costs, focus on discouraging unhealthy behaviors, such as physical inactivity, tobacco consumption, poor dietary habits, and stress & anxiety [4]. Health risk behaviors such as these are highly related to the most common chronic illnesses in the workplace, such as cardiovascular diseases, cancer, diabetes, cholesterol, and obesity, which are also the most preventable of all health concerns [6]. Health interventions in work environments are needed to help individuals change their health behaviors, yet companies often encounter difficulties along the way that impede the widespread implementation of such programs. One such obstacle is insufficient employee interest, especially among those workers with high-risk factors who would benefit most from participating [6].

Workplace health programs to encourage behavior change can embrace different types of interventions, from biometric screening to provide clinical measures of health, to wellness activities designed to promote healthy lifestyles through physical activity, healthier eating habits, and gambits to help stop smoking and manage stress [4]. Most participants engaged in health style programs mention the lack of motivational support to adopt and maintain lifestyle changes [24]. However, some of the obstacles encountered may come from the work environments themselves. A previous investigation revealed that workloads, temptations around the office and time constraints were reported as being the workplace obstacles most associated with a lack of engagement in adopting healthy lifestyles [15]. For that reason, it is particularly vital to not only understand how such factors in the workplace can influence workers’ health behaviors, but to also investigate their relationship with some key organizational variables [15]. Below, we explore some relationships between health behaviors and some organizational and individual outcomes.

### The Relationship Between Health, Productivity, Engagement and Mental Well-Being

There is abundant evidence in the literature that suggests individuals’ health and well-being are related to several productivity outcomes affected by both presenteeism and absenteeism [4, 38, 39]. Investing in workers’ health, by means of preventive interventions aimed at discouraging unhealthy behaviors may improve employees’ on-the-job performance and productivity. Since poor employee health is directly related to lower productivity [40], wellness programs targeting physical activity and nutrition have been applied in organizations and have yielded satisfactory results concerning productivity outcomes [41]. Many previous studies have shown that health-related problems are associated with higher absenteeism and presenteeism [41–43]. This means that besides the evident benefits for individuals, workplace health programs can result in productivity gains for companies. Social robots can be used in workplace health programs, with the additional advantage that they may be able to reduce the demands that contribute to presenteeism.

There is evidence in the field of human–robot interaction that individuals’ productivity can improve when a robotic agent is present, as opposed to situations with no robotic presence [44, 45]. As stated earlier, previous work has established that people can engage in empathetic and trustworthy relationships with robots because they are perceived as supportive, reliable, and tolerant [1]. These aspects may help to improve the intervention scores of the individuals followed by the robot agent. More specifically, this may result in better health-related outcomes, and consequently, higher productivity. Thus, we formulate the following hypothesis:

#### H1

The intervention with the robot agent will be associated with an improvement in individuals’ productivity despite sickness presenteeism at Time 2. Specifically, those participants will have post-test scores for productivity despite presenteeism, that are significantly higher than the scores of the group with the human agent.

Employee engagement has been an organizational variable conceptualized in several ways, all of which incorporate behavioral, cognitive and affective dimensions [46]. For this paper, we focused on workers’ emotional and behavioral reactions, analyzing both physical and emotional engagement. Physical engagement concerns the investment of effort, physical energy, and hard work with regard to task completion, whereas emotional engagement concerns emotional and affective reactions related to the work role itself [47].

Previous research [41] raised awareness of the relationship between health conditions and the correlations with employee engagement, and other investigations have clearly shown that work engagement is associated with a wide range of work and health outcomes which, in turn, are associated with workers’ increased quality of life and positive work-related behaviors [48]. Engagement has been conceptualized as an organizational variable with specific behavioral, cognitive and affective dimensions that help individuals commit to their work [49].

Overall, there has been a little effort to integrate work engagement in the literature on human–robot interaction. Indeed, the literature has so far revealed that individuals can become emotionally attached to robots, which may lead them to become more engaged in their tasks [50]. Since better health is related to greater engagement levels [48], we can consider that the health-intervention with the robot agent would improve individuals’ engagement levels and consequently improve post-intervention scores. We rely again on the assumption that the interaction with the robot agent will lead to better post-intervention scores than the intervention with the human agent, since social robots possess affective attributes that can help sustain people’s engagement, motivation, and provide social support [1]. The robot agent may also be a valuable resource for reducing the demands associated with presenteeism, such as in the case of work engagement [13]. For this reason we postulate:

#### H2

The intervention with the robot agent will be associated with an improvement in individuals’ engagement at Time 2. Specifically, those participants will have post-test scores for engagement significantly higher than the scores of the group with the human agent.

There is growing interest in the concept of mental well-being and its overall implications with regard to aspects of human life, including work-life aspects [51]. While it may be evident that physical activity, good dietary habits and no tobacco consumption can directly improve general well-being and quality of life [52], their association with mental well-being may not be particularly clear. Mental well-being is described as a complex construct focused on the subjective experience of life satisfaction, happiness, self-realization, and aspects of psychological functioning [51]. Indeed, being linked to some behavioral risk factors such as tobacco consumption, obesity, lack of physical activity and poor dietary habits [53], mental well-being has emerged as an important predictor of general health and longevity. Therefore, given that poor mental well-being is generally associated with physical diseases and unhealthy lifestyles, it has become an important public health concern [53].

Interestingly, a notable number of studies on human–robot interactions have revealed that social robots can assist people to easily improve their psychological outcomes [3, 22]. Social robots can provide comfort, listen without interrupting, and give support [24]. They are also seen as being free from the “social baggage” associated with human counsellors and therapists, making them appear nonjudgmental in a way that nurtures willingness to disclose [1]. Moreover, social robots can be used as a job resource (in the form of social support) that can assist individuals in some aspects of the psychosocial work environment that may have an impact on their mental health. For these reasons, we consider that the participants guided by the social robot will have better well-being levels at post-intervention scores than the group guided by the human agent. We hypothesized:

#### H3

The intervention with the robot agent will be associated with an improvement in individuals’ mental well-being level at Time 2. Specifically, those participants will have post-test scores for mental well-being significantly higher than the scores of the group with the human agent.

Overall, one of the main purposes of the present investigation is to compare the effect a health behavior intervention has on two distinct groups: one guided by a robotic agent and the other guided by a human agent. As can be seen from our hypotheses 1 to 3, we are interested in investigating what effect the type of agent has on each of the outcome variables. More specifically, we wish to determine whether the effect of the robotic agent is associated with better results for the outcome measures (productivity despite sickness presenteeism, engagement and mental well-being), or whether intervention guided by the robotic agent may be related to some outcomes but not others. For the last hypothesis, we intend to test whether the type of agent (robot or human) can influence post-intervention scores in all outcome measures:

#### H4

The type of agent will influence the levels of productivity despite presenteeism, the levels of engagement and the levels of mental well-being in the post-intervention scores.

## Methods

### Participants

Sixty-eight participants were recruited from two Portuguese organizations, from services and retail providers, and invited to participate in a health promotion program. Prior to the beginning of the study, the research team obtained written consent from participants. Ethical approval was gained from the ethics committees of the two universities involved in the investigation (code 69/2019 and code 17/2019), confirming that the robot’s behavior did not involve considerable risks for the participants. Specifically, the two ethical approvals were centered on participant anonymity, data confidentiality, informed consent, and the absence of risk for the participants during contacts with the robot or human agent. Additionally, the ethics committees at both universities believed that holding the debriefing at the conclusion of the study would lessen any discomfort the participants may have experienced throughout the sessions. Ten participants dropped out over the course of the sessions and two did not complete the final assessment at Time 2, resulting in a final dataset of fifty-six valid participants. From this fifty-six, thirty-seven were randomly assigned to the robot agent condition and nineteen were randomly assigned to the human agent condition. Concerning the participants’ distribution throughout the 4 health-related conditions, this was as follows: 23 subjects for the physical activity intervention (16 for the robot agent condition and 7 for the human agent condition), 18 for the nutrition habits (11 for the robot agent condition and 7 for the human agent condition), 4 subjects for tobacco consumption intervention (3 for the robot agent condition and 1 for the human agent condition), and 11 for stress and anxiety (7 for the robot agent condition and 4 for the human agent condition). This distribution was established at the end of the first session, because participants had the opportunity to confirm or modify the health-related focus of their participation during this session. The participants received no reward or compensation of any kind.

Ages ranged between 22 and 53 years old (*M* = 37.66; *SD* = 8.67). 74.5% of the sample were female and 25.5% were male. The majority of the participants were married (40%), or living in a non-marital partnership (29.1%). In terms of academic qualifications, the sample is highly qualified—56.4% reported to have at least completed an undergraduate degree. 92.6% also reported having a permanent contract with the company. Several independent sample *t* tests were performed in order to ensure there were no significant differences between the two groups concerning age (*t*_(48)_ = .677, *p* = .252), gender (*t*_(53)_ = − .926, *p* = .179) and academic qualifications (*t*_(53)_ = − .450, *p* = .327).

It is important to state that, for the robot agent condition, the research team asked whether each participant had previously interacted with a social robot. None of the participants said yes, this was the first experience for everyone.

### Materials—EMYS Robot (for the Robot Agent Condition)

**Fig. 1:**
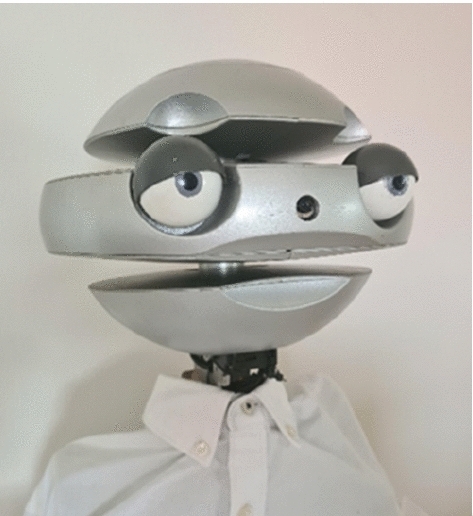
The EMYS robot used in the present investigation

The robot used in this research is EMYS, a social robot with a system designed to simulate certain features of the human mind (Fig. 1). Although EMYS can operate autonomously, it does not always need to act autonomously, sometimes it can be assisted by a human. In order to meet the investigations’ purposes, the research team chose to operate EMYS in a semi-autonomous system, so the dialogues and feedback provided by the robot could be more similar to those of a human.

EMYS has a head and no body, it can move its head, speak, and use certain facial expressions to connect with the user. The robot has been especially designed for human–robot interaction experiments. The paradigm used to operate the robot was the “Wizard of Oz” paradigm. This method involves simulating autonomy with a human "assistant", manipulating the robot's behavioral features, in particular, its speech. The EMYS robot is commonly applied in interventions in the field of human–robot interaction (e.g., [54, 55]). Although the participants believed the robot was completely autonomous, in reality, it was controlled by a researcher. The intention was that the participants would not realize this.

### Intervention Design and Content

The intervention lasted 3 months, from completion of the baseline assessment to completion of the post-intervention questionnaire. During the baseline assessment, each participant was able to choose a health-related behavior they would like their participation to focus on. There were four possibilities: physical activity, nutrition habits, tobacco consumption and stress and anxiety. Afterwards, they were randomly distributed between the two conditions (robot agent condition or human agent condition). During the first session with the agent (human or robot), participants had the chance to confirm or modify the health-related focus of their participation.

In total, each participant had eight sessions with the agent over eight weeks (one session of 20–30 min per week). Due to the impact of the COVID-19 pandemic, all the sessions occurred in a videoconference format, via Microsoft platform Teams. With the participants’ consent, all the sessions were recorded. In the final assessment, when responding to a question regarding the videoconference format of the investigation, 78.6% of the participants responded that the research format had not compromised their commitment to, or performance of the investigation. A *t* test was performed in order to ensure there were no differences between the participants who answered that the research format had not compromised their commitment and those who answered that it might have compromised it (*t*_(54)_ = .048, *p* = .481).

The intervention targeted multiple behavior change techniques that mapped onto the constructs in the motivational and volitional phases of the HAPA framework [8]. This socio-cognitive model aimed at describing, explaining and modifying health behaviors within individuals has been applied in several interventions, with good results in promoting health behavior change [16, 56]. In this intervention, participants were guided by the agents to set goals, monitor their behavior, elaborate action plans and coping strategies, and increase their self-efficacy by watching videos and reading testimonials. Specifically, throughout the eight sessions, the social agent (human or robot) targeted the following constructs: outcome expectancies (e.g., the agent encouraged the individuals to formulate their own potential pros and cons of the health behavior change); self-efficacy (e.g., instructions were given and effective behaviors were role-modeled); risk perceptions (e.g., the agent gave information about the existence of health risks); social influences (e.g., the agent asked individuals to demonstrate support for their co-workers who were also engaged in the intervention); action planning (e.g., individuals were asked to make concrete plans of when and how they should perform the health behavior, using the if-then formulation); coping strategies (e.g., individuals were asked to identify obstacles and possible solutions by developing coping strategies); and action control (e.g., the agent provides a digital calendar for individuals to monitor and indicate (daily or weekly) the times they practise the health behaviors). Besides that, at the beginning of each session the social agent discussed with the participants what they had done since the last session to move the plan forward and then provided appropriate feedback. This was the only content that was not standardized between both conditions and between the participants, because it had to be adapted to the experience of each participant and the actions that he/she developed throughout the week. This means that each session under both conditions (human agent or robot agent) was planned in advance by the research team to ensure that each participant could receive the right feedback regarding the health-behavior actions they had taken the week prior. For the robot agent condition specifically, multiple sentences were developed and added to the robot’s software. During the sessions, the human assistant only needed to select the most suitable response or question for the robot to display. For the human agent condition, the human agent was instructed to ask/response a specific set of several sentences that were highly comparable to the robot’s sentences, in order to maintain content similarity between the conditions. Regarding the seven constructs that were the focus of the study (outcome expectancies; self-efficacy; risk perceptions; social influences; action planning; coping strategies and action control), all participants were exposed to them in the exact same sessions, whether being followed by the human agent or the robot agent.

The datasets generated during the current study are available from the corresponding author upon reasonable request.

### Measures

All assessments listed below were self-report measures that were completed online in response to emails that included links to the surveys. This approach allowed participants to complete the assessments in their own time to reduce the research burden, and allowed the assessments to be both independent of the research team and separate from the intervention with the social agent. All measures were assessed prior to the intervention (Time 1) and one month after the intervention (Time 2). At both times, all measures presented satisfactory internal consistency.

### Productivity Despite Presenteeism

Productivity despite presenteeism was measured using an adaptation of the original version of the Stanford Presenteeism Scale (SPS-6), developed by Koopman et al. [57]. The SPS-6 measures individuals’ capacity to complete work tasks and avoid distraction. Examples of the items include “I would feel desperate with regard to accomplishing certain tasks” and “My job would be much harder to handle”. The Likert scale ranges from 1- strongly disagree to 5- strongly agree. Cronbach’s α was .81 at Time 1, and .87 at Time 2.

### Work Engagement

Engagement was measured on a 12-item scale [58] designed to measure two global dimensions of the engagement construct, namely emotional engagement and physical engagement. Emotional engagement assesses the extent to which people experienced positive feelings about their work in general (e.g., “I am proud of my work”), and physical engagement assesses to what extent they invested physical energy and effort in their task (e.g., “I have devoted a lot of energy to my work.”). This scale ranges from 1- never to 5- always. Cronbach’s α was .93 at Time 1 and .92 for Time 2.

### Mental Well-Being

Mental well-being was measured in accordance with the Warwick-Edinburgh Mental Well-being Scale [51] comprising 14 items that evaluate mental well-being in the general population, and covers both the feelings and functioning aspects of mental well-being. Examples of the items include “I’ve been dealing with problems well” and “I’ve been feeling optimistic about the future”. The scale ranges from 1 (never) to 5 (always), where higher levels are associated with better mental well-being. Cronbach’s α was .91 at Time 1 and .92 for Time 2.

## Results

All analyses were performed using SPSS version 26. Descriptive statistics and correlations among variables can be found in Table 1.

**Table 1:** Descriptive statistics and correlations among studied variables

Variables	N	M	SD	Correlations				
				1	2	3	4	5
1. Productivity despite presenteeism T1	43	3.17	0.45					
2. Work Engagement T1	54	4.07	0.56	.064				
3. Mental well-being T1	55	3.53	0.55	− .069	.506**			
4. Productivity despite presenteeism T2	48	3.40	0.85	− .151	.403**	.308*		
5. Work Engagement T2	52	4.08	0.52	.010	.678**	.276*	.380*	
6. Mental well-being T2	55	3.83	0.54	.196	.277*	.323*	.268	.170

*SD*  standard deviation

**p < .01; *p < .05

Concerning the first hypothesis, which predicted that the use of the robot agent would improve participants’ productivity despite sickness presenteeism at Time 2, we can observe that for the robot agent group, the post-test mean levels of productivity were significantly higher than at pre-test (M_T1_ = 3.19, M_T2_ = 3.73, *t*_(1,46)_ = 9.041, *p* < .001, d = .89). For the human agent group, levels of productivity despite sickness presenteeism at Time 2 were not statistically different from those at Time 1 (M_T1_ = 3.12, M_T2_ = 2.82, *t*_(1,41)_ = .05 *p* = .62, d = .59). We tested our first hypothesis by comparing the change in productivity despite sickness presenteeism of the robot agent group with the change that occurred in the productivity despite sickness presenteeism of the human agent group at the same assessment moments, which was from the pre-intervention measurement at T1 to the T2 measurement (post-intervention measurement). In a paired *t* test, the difference in change in productivity despite sickness presenteeism for the two groups was significant (t_(47)_ = − 25.953, *p* < .001). To understand the relationship between the type of agent and individuals’ productivity despite presenteeism at Time 1 versus Time 2, a repeated-measures ANOVA was performed. The results revealed that the type of agent had a significant effect on the productivity levels (*F*_(1, 46)_ = 9.041, *p* < .005; η^2^ = .26). These results support our first hypothesis by showing that the intervention with the robot agent was effective in increasing participants’ productivity at Time 2.

Regarding the second hypothesis, it predicted that the use of the robot agent would be associated with a positive change in participants’ engagement level at Time 2. Thus, our expectation was that the scores of the participants’ engagement would change significantly from Time 1 to Time 2. A *t* test showed that there were no differences in the levels of engagement between Time 1 and Time 2 for either the robot agent condition (M_T1_ = 4.21, M_T2_ = 4.12, *t*_(33)_ 1.161, *p* > .05), or the human agent condition (M_T1_ = 3.87, M_T2_ = 4.05, *t*_(18)_ − 1.722, *p* > .05). To test whether there were significant differences between the type of agent and the participants’ engagement level at Time 1 and Time 2, a further analysis of these results was carried out using a repeated measures ANOVA. The results showed that there were no significant differences (*F*_(1, 49)_ = .5176, *p* > .005). Overall, these results do not corroborate hypothesis 2.

Our third hypothesis predicted that the intervention with the robot agent would be associated with a positive change in participants’ mental well-being level at Time 2. Thus, our expectation was that the scores of the participants’ mental well-being would change significantly from Time 1 to Time 2. Moreover, we expected that this change would be greater for the robot agent condition than the one observed for the human agent condition. The post-mean levels determined that the mean scores for mental well-being differed significantly across the two assessment moments for the robot agent condition (M_T1_ = 3.57, M_T2_ = 3.99, *t*_(37)_ = − 4.130, *p* < .001, d = .61). For the human agent condition, the mean scores were not statistically significant (M_T1_ = 3.46, M_T2_ = 3.50, *t*_(18)_ = .408, *p* > .005, d = .56). In a paired *t* test, the difference in change in mental well-being for the two groups was significant (t_(54)_ = − 3.412, *p* < .001). This means that participants in the robot agent condition showed higher levels of well-being at Time 2 than the participants in the human agent condition. To understand the relationship between the type of agent and individuals’ well-being at Time 1 versus Time 2, a repeated-measures ANOVA was performed. The results revealed that the type of agent had a significant effect on the well-being levels (*F*_(1, 53)_ = 4.517, *p* < .005; η^2^ = .079). These results support our third hypothesis by showing that the intervention with the robot agent was effective in increasing participants’ well-being levels.

**Fig. 2:**
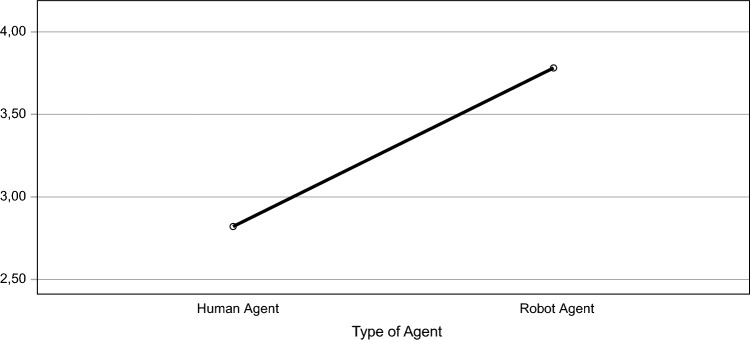
Effect of the type of agent on productivity despite presenteeism level

**Fig. 3:**
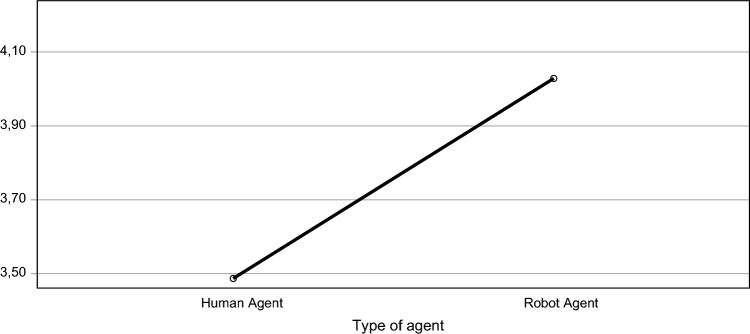
Effect of the type of agent on mental well-being level

Regarding our fourth hypothesis, which predicted that the type of agent would influence the levels of productivity despite sickness presenteeism, mental well-being and engagement in the post-intervention scores, a One-Way MANOVA was performed, with the type of condition included as a covariate. There was a statistically significant difference in the outcome variables based on the type of agent (*F*_(1, 43)_ = 8.997, *p* < .001, Wilk’s Λ = .597, partial η^2^ = .40). In particular, the type of agent had a statistically significant effect on productivity despite presenteeism post-intervention scores (*F*_(1, 45)_ = 17.628, *p* < .001; partial η^2^ = .29) (Fig. 2), and on mental well-being post-intervention scores (*F*_(1, 45)_ = 11.009, *p* = .002; partial η^2^ = .20) (Fig. 3), supporting *H4* for these variables. No statistically significant differences were found between the type of agent and engagement post-intervention scores (*F*_(1, 45)_ = .872, *p* = .352; partial η^2^ = .02).

## Discussion

This longitudinal investigation compared the efficacy of a robot agent with that of a human agent with regard to promoting health in work contexts. The main focus of the study was to analyze which type of agent would be associated with better results in a set of individual and organizational outcomes. Our results showed that the intervention with the robot agent was associated with improvements in individuals’ productivity despite sickness presenteeism and well-being levels. However, neither type of agent had any effect on the engagement levels of the participants.

Thus, demonstrating the potentialities of using social robots to establish therapeutic and worth relationships with employees in their workplaces, while improving their health status, constitutes interesting new findings for the health behavior change literature [7, 17], the HAPA theoretical framework [8], the JD-R approach to presenteeism [12, 13], and the literature on human–robot interaction [25]. Furthermore, our results may also contribute to the presenteeism literature, by demonstrating the potentialities of a health promotion program using social robots to enhance productivity despite presenteeism. This may lead to an increase in workers’ productivity and well-being levels, and consequently an improvement in related health and quality of life. Below, we explore in more detail the theoretical and practical implications of this research.

### Theoretical and Practical Implications

The results of our research extend previous findings from the health behavior change literature and the HAPA framework, and their application in the context of technology-driven interventions [5, 22, 24, 25]. Furthermore, there are potential advantages to using social robots over other technology-delivered applications in workplace contexts, it being suggested that robots can encourage health behavior change in individuals as well as engage in therapeutical relationships with individuals [24]. This could be especially relevant for scenarios such as medical and therapy settings, by demonstrating that even when there is no human interaction, the use of robots may not compromise individuals’ health-related outcomes. Moreover, this investigation also contributes to the JD-R model, by showing that social robots can be used in workplace interventions to reduce job demands associated with presenteeism [13], helping workers to adopt healthier behaviors. Specifically, social robots may be powerful resources in workplaces when employees are facing higher job demands (whether because of task characteristics or because of their personal health status). This support given by robots can be cognitive support, motivational or physical collaboration, as stated previously by [14]. The results of this same investigation have shown that social robots have the potential to be more readily accepted than a human colleague in situations where individuals are working ill. Our investigation is in line with the goals and results of previous research on an attempt to determine the potential possibilities of social robotics for tackling presenteeism. Our research acknowledged that individuals are open to taking on board the recommendations of a social robot concerning health-related issues, and this may improve their productivity despite presenteeism. In general, we argue that social robots may possibly be used in workplaces not to substitute human agents or therapists, but to reduce workloads and job demands.

As previously stated, the health-promoting intervention with the robot was not associated with improvements in engagement levels. Although we did not expect these results, they are in line with some previous research in healthcare settings, where it is usual to find better results when a robot is used to complement or mediate the relationship between the therapist and the patient, and to mediate the activities of the therapist [22]. Following this line, our study’s findings can also contribute to improve the quality of training delivered by human agents in healthcare interventions. Similarly, other recent studies advance that robots can be a viable way to raise awareness of health education and health behaviour change, but their full integration into the clinical process may not be required [25]. Although this might be a possible explanation, engagement has not been as thoroughly studied in human–robot interaction research [59], especially in workplace scenarios. To the best of our knowledge, this investigation constitutes one of the first attempts to link social robots to some organizational outcomes such as work engagement, so additional research on human–robot interaction within work environments is needed, as mentioned by previous authors [35, 59]. Such further research would likely give rise to interesting new findings regarding the advantages of using social robots within workplaces.

Our findings that the intervention guided by the robot led to improvements in participants’ productivity despite presenteeism, and in their mental well-being are in line with a previous body of research that links human–robot interaction interventions with better productivity outcomes and individuals’ psychological well-being [3, 22, 50]. This evidence that the power of artificial intelligence machines can be harnessed to deliver health interventions that promote employee productivity and well-being contributes valuable information to the presenteeism literature.

Based on our current findings, we can recommend that practitioners and managers embrace the use of social robots in work environments. In line with the literature [35, 50], there are clearly plenty of opportunities to test the implementation of social robots in workplaces. Even while not completely independent and working autonomously, social robots can complement interventions with practitioners [26], and thus contribute to the productivity and vitality of the workforce. Even from the point of view of a manager or a therapist, social robots may help to reduce the workload for humans, while assisting individuals to reach their goals and improve their health and quality of life [22]. They can engage people of all ages in deeply personalized experiences, attending to their health needs and goals [1] to induce them to accept and follow recommendations to improve their health and well-being levels. Furthermore, the introduction of social robots as health promoting agents within workplaces may help managers to deal with the phenomena of presenteeism and absenteeism, both of which can have such a high cost for companies [43].

Organizations can gain a competitive advantage from engaging in health-promoting programs, especially in light of the current worldwide labour shortages exacerbated by the COVID-19 pandemic. This study provides a solid argument for companies to implement interventions using social robots to help create a healthy, productive, and resilient workforce.

### Limitations and Directions for Future Research

We acknowledge some limitations of the study. First, participation in this research was voluntary, which means that we may not have reached the workers who could actually benefit the most (i.e., individuals with higher health-related risk factors). Unhealthy employees may gain the most from participating in this type of health promotion program, and yet they are less likely to engage in these interventions [6]. By the same token, the voluntary nature of this research means that this sample is not representative of all employees who may or may not be interested in health behavior change. Furthermore, since each participant had the option to select the focus of their health behavior intervention, there is the possibility of bias in the subject distribution of the four health behavior interventions.

Upcoming research should focus on performing a similar health-promoting intervention, particularly one involving artificial intelligence machines, on a larger sample. This should lead to firm conclusions about changes in health outcomes resulting from long-term interactions with a social robot.

A further weakness concerns the Wizard-of-Oz method applied in this research. Using the Wizard-of-Oz method raises concerns regarding the social deception and making the robot more like a human proxy without full autonomy than an autonomous machine [60]. However, previous studies have suggested the clear strengths of this method: it allows the robot to execute more complex actions in its interactions and dialogues with people; individuals can imagine what future interactions with robots will be; and it allows researchers to test design and communication features [60]. Nevertheless, future research needs to focus on the interaction between autonomous robot agents and individuals [35] in contexts of health intervention programs.

Moreover, research in human–robot interaction is particularly vulnerable to the novelty effect [61], thus we cannot rule out the possibility that this occurred in our study. Specifically, since it was the first time that all the participants were interacting with a robot, they may have behaved differently than they would normally do. This may be a possible explanation as to why the robot agent condition had better results than the human agent condition. Upcoming investigations must include a control mechanism to prevent the novelty effect, similar to the work of [62]. Additionally, it may be possible that the Hawthorne effect may have also occurred. In an effort to overcome these constraints, future health-promoting interventions including social robots might include a second group of participants that interact with some other form of novel element instead of the robot, such as a serious game.

Lastly, due to the COVID-19 pandemic situation and the adoption of remote work by most organizations, this intervention was undertaken in a non-presential context, where each participant had their sessions with the social agent in a videoconference format, instead of face-to-face interaction. We can relate this to the small sample size of our research, which limits the generalizability of the findings. We believe that if workers could interact face-to-face with the robot, they would be more interested in our research. Thus, if possible, the researcher intends to conduct this investigation in a presential context with another set of participants. This would allow a comparison of the differences between interventions performed presentially and non-presentially and, we hope, produce interesting data to report.

## Conclusion

This investigation compared the impact of a health behavior change intervention guided by two types of social agents (a human agent and a robot agent) on a set of organizational and individual outcomes. The results show that the robot agent was associated with better post-intervention scores in individuals’ productivity and mental well-being despite sickness presenteeism. Although these are preliminary results, they nevertheless show that robots can be used to provide virtual support for health behavioral change. At this critical junction, where the pandemic crisis caused by the COVID-19 virus is forcing long-distance relationships like remote work and tele-consulting, robotic partners may provide a great opportunity to enhance social interactions and improve people’s health outcomes.
